# Genetic diversity of calcareous grassland plant species depends on historical landscape configuration

**DOI:** 10.1186/s12898-017-0129-9

**Published:** 2017-04-24

**Authors:** Christoph Reisch, Sonja Schmidkonz, Katrin Meier, Quirin Schöpplein, Carina Meyer, Christian Hums, Christina Putz, Christoph Schmid

**Affiliations:** 1 Institute of Plant Sciences, University of Regensburg, 93040 Regensburg, Germany; 2 German Research Center for Environmental Health, Research Group Comparative Microbiome Analysis, Ingolstädter Landstr. 1, 85764 Neuherberg, Germany

**Keywords:** AFLP, Dry grasslands, Habitat fragmentation, Genetic diversity, Grazing, Land use, Litter, Soil analysis

## Abstract

**Background:**

Habitat fragmentation is considered to be a main reason for decreasing genetic diversity of plant species. However, the results of many fragmentation studies are inconsistent. This may be due to the influence of habitat conditions, having an indirect effect on genetic variation via reproduction. Consequently we took a comparative approach to analyse the impact of habitat fragmentation and habitat conditions on the genetic diversity of calcareous grassland species in this study. We selected five typical grassland species (*Primula veris*, *Dianthus carthusianorum*, *Medicago falcata*, *Polygala comosa* and *Salvia pratensis*) occurring in 18 fragments of calcareous grasslands in south eastern Germany. We sampled 1286 individuals in 87 populations and analysed genetic diversity using amplified fragment length polymorphisms. Additionally, we collected data concerning habitat fragmentation (historical and present landscape structure) and habitat conditions (vegetation structure, soil conditions) of the selected study sites. The whole data set was analysed using Bayesian multiple regressions.

**Results:**

Our investigation indicated a habitat loss of nearly 80% and increasing isolation between grasslands since 1830. Bayesian analysis revealed a significant impact of the historical landscape structure, whereas habitat conditions played no important role for the present-day genetic variation of the studied plant species.

**Conclusions:**

Our study indicates that the historical landscape structure may be more important for genetic diversity than present habitat conditions. Populations persisting in abandoned grassland fragments may contribute significantly to the species’ variability even under deteriorating habitat conditions. Therefore, these populations should be included in approaches to preserve the genetic variation of calcareous grassland species.

**Electronic supplementary material:**

The online version of this article (doi:10.1186/s12898-017-0129-9) contains supplementary material, which is available to authorized users.

## Background

Calcareous grasslands are important hotspots of plant species diversity in central Europe. They contain many rare and endangered plant species and are of strong conservation interest [1–3]. However, due to land use changes, calcareous grasslands declined significantly in Europe during the last 150 years [4]. Agricultural intensification, increased fertilization and afforestation caused a drastic loss of grasslands. In some regions, up to 90% of the grasslands disappeared [5]. Today calcareous grasslands are thus often highly fragmented, with the area of the grassland patches continuously decreasing while their spatial isolation increases [6]. This process of habitat fragmentation is a general threat to biodiversity, reducing species richness within small and isolated habitat patches [7].

However, fragmentation has not only an impact on biodiversity at the species level, but also on the genetic diversity, due to geographic isolation of smaller populations [8]. In particular formerly widespread species are more susceptible to the effects of fragmentation than naturally rare and isolated species [9]. Genetic variation is directly related to population size [10] and often decreases during the process of fragmentation. Moreover, the exchange of pollen and seeds between populations is impeded by fragmentation, which decreases gene flow [11] and increases genetic drift. This results in a loss of genetic diversity and may lead to reduced generative [12] and vegetative performance [13]. As a consequence, there is an increase in the susceptibility to pathogens and herbivores in the short term [14] and the probability of extinction in the long term [8]. Finally, the loss of genetic diversity may explain the observed loss of species diversity in numerous studies analysing fragmentation effects [6].

However, the results of many studies dealing with the impact of fragmentation on genetic diversity are inconsistent. Some of these studies support the assumptions derived from the theory of island biogeography [15], whereas others do not [16]. This dilemma has recently been illustrated by a review of 259 fragmentation studies, which concluded that the broad generalisations on the effects of fragmentation are problematic [17].

One of the most important challenges of fragmentation studies is that habitat conditions may differ between remnant grassland patches [17]. Many calcareous grasslands have been abandoned because sheep grazing is no longer economical [1, 2]. In the absence of grazing, habitat conditions continuously deteriorate and typical open short-grass conditions get lost [18]. This process is enhanced by the spill-over of fertiliser from adjacent agricultural areas [18]. Absence of grazing and increased nitrogen deposition change the vegetation structure of calcareous grasslands [19], resulting in a decrease in species richness [20] and shifts in species composition [21]. Due to the lack of biomass removal and increasing productivity litter accumulates while gaps of open soil, which are indispensable for the germination of many dry grassland species, become rare [22]. Furthermore, litter acts as a trap, further reducing the number of germinating seeds [23]. Particularly for species which require light to germinate, ground shadowing caused by increasing vegetation height, dominance of grasses and litter accumulation leads to the regression those of species [24]. Therefore, successful reproduction is impeded when habitat conditions deteriorate, which may subsequently affect genetic diversity. This process is intensified by the loss of suitable dispersal vectors, such as migrating sheep, which also reduces the exchange of seeds [2] and subsequently, the immigration of genetically deviant individuals.

Hence, genetic diversity may be affected by the interfering effects of both fragmentation and habitat conditions, which might explain the inconsistent results of many fragmentation studies. Moreover, due to different biological traits, patterns of genetic variation differ between plant species [25] and these may react completely different to fragmentation, again explaining unclear results of many genetic fragmentation surveys.

In this study, we analysed the impact of fragmentation on genetic diversity of several calcareous grassland species in a comparative approach, including both habitat fragmentation and habitat conditions. We used the same analytical approach for all species and applied Bayesian multiple regressions, which enables a detailed interpretation of the data, while being more flexibly adaptable to the data structure than traditional frequentist methods. More specifically we ask the following questions: (i) has fragmentation of calcareous grasslands a significant impact on genetic diversity of plant populations? (ii) is historical landscape configuration more important for genetic diversity than present landscape configuration? (ii) is genetic diversity of grassland plant populations affected by habitat conditions?

## Methods

### Study sites, habitat fragmentation and habitat conditions

**Fig. 1:**
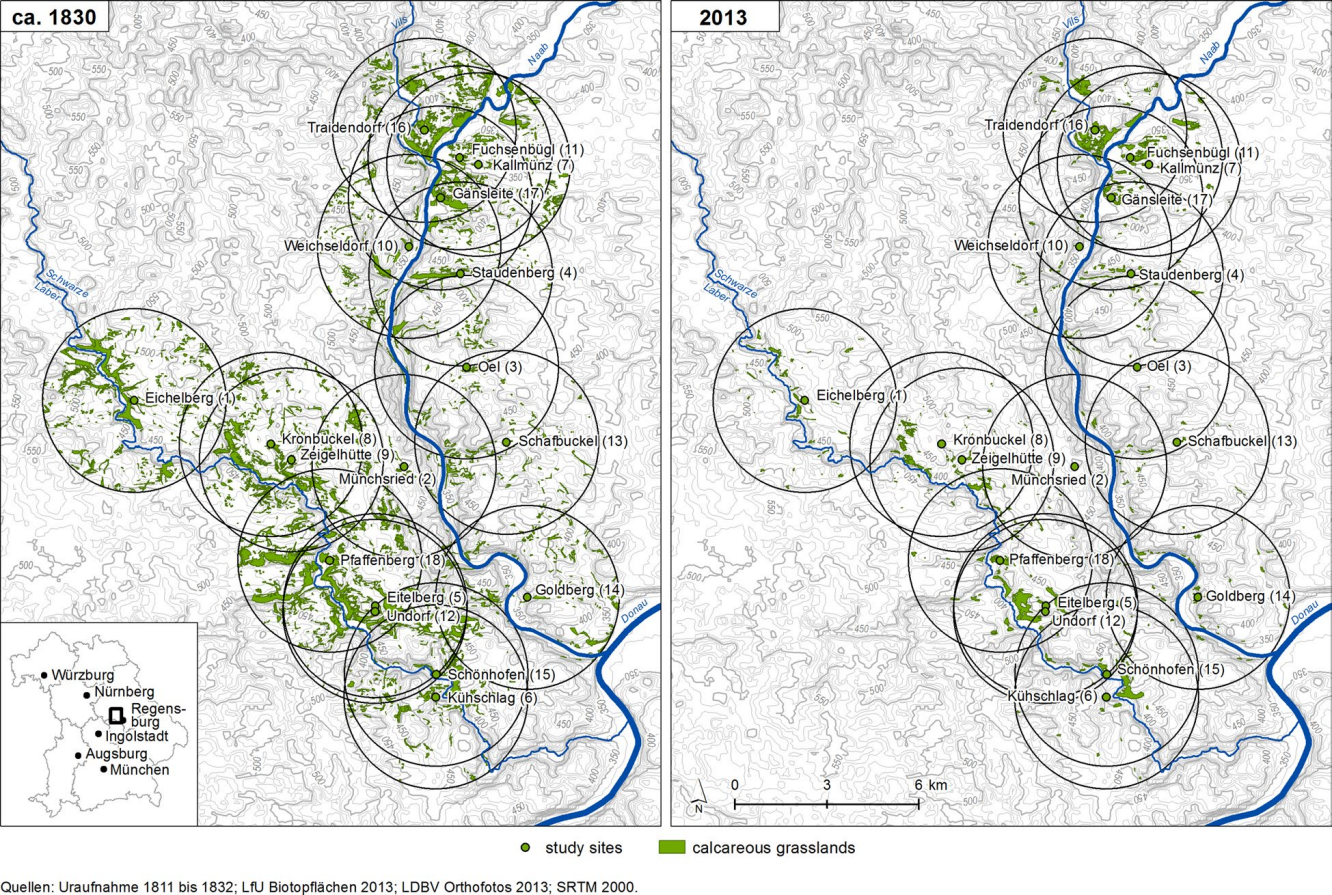
Geographic location of the 18 selected study sites (*labelled dots*) in the valleys of Naab and Laber on the Franconian Alb in south eastern Germany near Regensburg and all other calcareous grasslands (*grey areas*) within a radius of 3 km around the study sites in 1830 and 2013

For our study, we randomly selected 18 remnant calcareous grasslands in the valleys of the rivers Naab and Laber on the Franconian Alb in south eastern Germany near Regensburg (Fig. 1). Within the study region, calcareous grasslands have been subjected to fragmentation due to afforestation, intensification and abandonment since the nineteenth century. The study sites and all other calcareous grasslands occurring within a radius of 3 km around these sites were vectorised using a Geographic Information System (Arc Info 10.0, Esri) based upon corrected aerial photos (orthophotos) from August to October 2013 to study the effects of this fragmentation process. Vectorised data was used to calculate the current area (HA_2013_) and perimeter (P) of each grassland fragment as well as the distance (D_2013_) to the nearest calcareous grassland within the 3 km radius. The shape of the study sites was characterised by the ratio of habitat area to perimeter (HA/P_2013_), which was small for narrow and elongated grasslands and large for round and compact grasslands.

Using historical cadastral maps, which were available from local land surveying offices, we determined the area of the study sites (HA_1830_) and the distance (D_1830_) to the nearest calcareous grassland within the 3 km radius in 1830. The maps include a detailed legend, which allows the identification of calcareous grasslands. We compared the area covered by calcareous grasslands in 1830 and 2013 and calculated the habitat loss (HL) within the 3 km radius around each of our study sites as a percentage of the grassland area lost since 1830. Additionally, connectivity of each fragment to all other fragments within the 3 km radius in 2013 and 1830 was calculated according to Hanski [26] as $Si = \sum\nolimits_{{j \ne i}} {\exp \left( { - \upalpha d_{{{\text{ij}}}} } \right)A_{{\text{j}}} }$ where *Si* is the connectivity of the patch *i*, d_*ij*_ is the distance between patches *i* and *j*, and A*j* is the area of the patch *j* [27]. Based upon the historical maps, grasslands were classified for further analyses as historically old, when they were already grassland in 1830, and historically young grasslands, when they originated after 1830.

The selected grasslands date back at least to the period of the Roman Empire [28] and have been grazed frequently until the 1960s, as have most other grasslands in central Europe [4]. Today they are abandoned or infrequently grazed. However, detailed information about the grazing history since the 1960s is not available. In 2014, we established ten study plots with the size of 2 × 2 m at each of the selected grasslands to analyse the impact of the habitat conditions on genetic diversity of our study species. In each plot we determined the vegetation height (VH) as well as the cover of grass (CG), litter (CL) and bare soil (BS). Furthermore, we took five soil samples at each study site with a core sampler, which were then pooled, in order to analyse the nutrient content of the soil. Pooled samples were dried in a heating cabinet at 50 °C for several days, cleaned by sieving with 2 mm mesh size and then stored at 4 °C until they were subjected to a soil chemical analysis following the procedures described by Bassler et al. [29]. We determined the phosphorous (P) and potassium (K) content, as well as the carbon to nitrogen ratio (C/N) as described previously [30].

### Study species and genetic variation

For our study we selected five typical and widespread calcareous grassland species (*Primula veris* L., *Dianthus carthusianorum* L., *Medicago falcata* (L.) Arc., *Polygala comosa* Schkuhr and *Salvia pratensis* L.), frequently occurring in calcareous grasslands in south eastern Germany. In the field we assessed population size (NI) by counting the number of present individuals (Table 1) at each site. For the analysis of genetic variation with a few exceptions (at three sites *P. comosa* could not be sampled), leaf material of 15 individuals per population and species was collected (Table 5). In total 1286 individuals from 87 populations were analysed. Leaf material was placed in plastic bags in the field and stored in a lab freezer at −20 °C until molecular analysis.

**Table 1:** Size of the studied populations

St.	Name	*P.v.*	*D.c.*	*M.f.*	*P.c.*	*S.p.*
01	Eichelberg	138	24	238	138	433
02	Münchsried	782	266	67	782	458
03	Oel	110	196	110	110	1057
04	Staudenberg	30	13	23	30	41
05	Eitelberg	145	302	415	145	287
06	Kühschlag	429	265	57	429	844
07	Kallmünz	120	741	351	120	487
08	Kronbuckel	1288	146	166	1288	1251
09	Ziegelhütte	1173	972	246	1173	1565
10	Weichseldorf	421	1790	905	421	740
11	Fuchsenbügl	61	7115	504	61	1140
12	Undorf	630	69	1348	630	735
13	Schafbuckel	7690	2757	784	7690	2312
14	Goldberg	143	2391	1809	143	3319
15	Schönhofen	380	368	702	380	3735
16	Traidendorf	2694	4097	401	2694	8410
17	Gänsleite	2707	4310	1174	2707	18,760
18	Pfaffenberg	12,938	8125	9457	12,938	53,585

Population size of *Primula veris* (*P.v.*), *Dianthus carthusianorum* (*D.c.*), *Medicago falcata* (*M.f.*), *Polygala comosa* (*P.c.*) and *Salvia pratensis* (*S.p.*) at the study sites determined as the number of occurring individuals

Genomic DNA was isolated from dry leaf material using the CTAB-based method [31] as described before [32]. Concentrations of the DNA extracts were measured photometrically. DNA solutions were diluted with water to 7.8 ng/μL and used for the analysis of Amplified Fragment Length Polymorphisms (AFLPs), which were conducted concordant with the protocol from Beckmann Coulter as described previously [33, 34].

DNA adapters were prepared by adding equal volumes of both single strands of *Eco*RI (4 µM) and *Mse*I (40 µM) adaptors (MWG Biotech), following a 5 min heating at 95 °C with a final 10 min step at 25 °C. DNA restriction and adapter ligation were performed in one step by adding a 3.6 μL mixture per reaction containing 2.5 U *Eco*RI (MBI Fermentas), 2.5 U *Mse*I (MWG Biotech), 0.1 μM *Eco*RI and 1 μM *Mse*I adapter pair, 0.5 U T4 Ligase with 0.1× of its corresponding buffer (MBI Fermentas), 0.05 M NaCl and 0.5 μg BSA (New England BioLabs) to 6.4 μL of genomic DNA in a concentration of 7.8 ng/μL. Following an incubation at 37 °C for 2 h with a final enzyme denaturation step at 70 °C for 15 min, the restriction-ligation products were diluted tenfold with 1× TE buffer for DNA (20 mM Tris–HCl, pH 8.0; 0.1 mM EDTA, pH 8.0).

For preselective DNA amplification, 1 μL diluted DNA restriction-ligation product, 0.25 µM preselective *Eco*RI and *Mse*I primers (MWG Biotech) were added to an AFLP Core Mix (PeqLab, Germany) containing 1x Buffer S, 0.4 mM dNTPs and 1.25 U/µL Taq-Polymerase. In a 5 μL reaction volume PCR was performed on at 94 °C for 2 min then 30 cycles of 20 s denaturation at 94 °C, 30 s annealing at 56 °C and 2 min elongation at 72 °C, a final 2 min 72 °C and 30 min 60 °C step for complete extension ending with a final cool down to 4 °C. After PCR, products were diluted 20 fold with 1× TE buffer for DNA.

After an extensive screening of 30 primer combinations, three primer combinations per species were chosen for a subsequent selective PCR reaction. For detection, *Eco*RI primers labelled with different fluorescent dyes (Beckman Coulter) were used (Table 2). Selective PCR was carried out in a total reaction volume of 5 μL containing an AFLP Core Mix (1× Buffer S, 0.4 mM dNTP’s, 1.25 U/µL Taq-Polymerase, PeqLab, Germany), 0.05 μM selective *Eco*RI (Proligo, France), 0.25 μM *Mse*I (MWG Biotech) primers and 0.75 μL diluted preselecive amplification product. For detection, *Eco*RI primers labelled with different fluorescent dyes (D2, D3, D4) were used. PCR parameters used were treated 2 min at 94 °C, 10 cycles 20 s denaturation at 94 °C, annealing 30 s at 66 °C and 2 min elongation at 72 °C, where annealing temperature was reduced every subsequent step by 1 °C, additional 25 cycles of 20 s denaturation at 94 °C, 30 s annealing at 56 °C and 2 min elongation at 72 °C completed by a following 30 min step at 60 °C and a cool down to 4 °C.

**Table 2:** Selective primer pairs used for AFLP analysis of the study species

Species	D2	D3	D4
*Primula veris*	CAA-AAC	CAA-ACG	CAG-ACA
*Dianthus carthusianorum*	CTC-AGC	CAA-AAG	CTG-ACT
*Medicago falcata*	CAC-ACC	CTA-ACG	CTT-ACA
*Polygala comosa*	CAA-AAC	CAT-ACG	CTA-ACA
*Salvia pratensis*	CTT-AGC	CTA-AGG	CTT-ACA

Selective PCR products were diluted with 1× TE_0.1_ buffer for AFLP and pooled. After pooling 5 μL of each selective PCR product of a given sample and adding them to a mixture of 2 μL sodium acetate (3 M, pH 5.2), 2 μL Na_2_EDTA (100 mM, pH 8) and 1 μL glycogen (20 mg/mL; Roche), DNA was precipitated in a 1.5 mL tube by adding 60 μL of 96% ethanol (−20 °C) and an immediate shaking. DNA was pelleted by 20 min centrifugation at 14,000*g* at 4 °C, the supernatant was poured off and the pellet was washed once by adding 200 μL 70% ethanol (−20 °C) and centrifugation at the latter conditions and was subsequently vacuum dried in a concentrator [33, 34].

After redissolving the pelleted DNA in a mixture of 24.8 μL Sample Loading Solution (SLS, Beckman Coulter) and 0.2 μL CEQ Size Standard 400 (Beckman Coulter), selective PCR products were separated by capillary gel electrophoresis on an automated sequencer (GeXP, Beckmann Coulter). Results were examined using the GeXP software (Beckman Coulter) and analysed using the software Bionumerics 4.6 (Applied Maths, Kortrijk, Belgium). From the computed gels, only those fragments that showed intense and articulate bands were taken into account for further analyses. Samples yielding no clear banding pattern or obviously representing PCR artefacts were repeated. Finally, 19 individuals were excluded from the analysis due to the lack of a clear banding pattern. Reproducibility of molecular analyses was investigated with 10% of all analysed samples by means of estimating the genotyping error rate [35], which was 3.8%.

From the AFLP bands, a binary (0/1) matrix was created for each species. Based upon this matrix, we calculated the genetic diversity of each population as Nei’s Gene Diversity [36] using the program AFLP SURV [37].

### Bayesian multiple regressions

The impact of habitat fragmentation and habitat conditions on diversity was analysed using a robust hierarchical Bayesian multiple regression approach with regression parameters for the species level (not shown) and an overarching set of hyperparameters for the species-independent estimation of regression parameters. Predictor variables were grassland fragmentation parameters (fragment area, area/perimeter ratio as well as distance to the nearest calcareous grassland and habitat connectivity) and habitat condition parameters (vegetation height, cover of grass, litter and bare soil, contents of P, K and C/N ratio). This approach is equivalent, though not identical, to a linear mixed model with random slopes and random intercepts using species as a random effect. The overarching hyperparameters ensure a transfer of information between the species’ parameters that shrinks outliers on species-level towards the main trend. The hyperparameters themselves are therefore suitable to depict the species-independent trends in the data.

There was no credible influence of habitat age detectable by the model and, hence, impeded accurate parameter estimation of the remaining parameters. Accordingly, habitat age as a parameter hampered model interpretation, and therefore was excluded from the final analysis. All predictor variables were checked for strong correlations (Pearson’s correlation coefficient >0.8; observed maximum 0.7) to check for multicollinearity. Additionally, multicollinearity can be recognised in Bayesian models by extremely broad posterior distributions of correlated parameters. However, this was not the case in the presented analysis. Model stability was verified by re-running the analysis with and without the most strongly correlated predictors. Accordingly, habitat age as a parameter hampered model interpretation and was, therefore, excluded from the final analysis.

A Bayesian approach was chosen for being flexibly adjustable to the situation at hand, e.g. it can be easily modified to reduce false positives in parameter estimation or improved to accommodate outliers in the data. In the situation at hand, the applied model could easily be tailored to reflect the hierarchical structure of our data (Additional file 1). Furthermore, results from Bayesian models have a higher informative value than classical NHST methods as they provide full probability distributions on the estimated parameters. Modelling and interpretation were carried out using the software packages R 3.2.1 [38] and JAGS 3.2.0 for Markov Chain Monte Carlo (MCMC) sampling [39] as well as utility functions provided by Kruschke [40]. Errors were modelled as being t-distributed in order to accommodate outliers and conduct a robust regression. Regression parameters were regularised using mildly informed, double-exponential prior distributions with location parameter set to 0 and a fixed precision parameter set to 0.1, thereby reducing chances for false positive regression parameters. These settings, known as the Bayesian Lasso [41] avoid overfitting in complex models and reduce the overestimation of effects that can happen in AIC-based model selection procedures.

Sampling was carried out with four MCMC chains with 300,000 steps in total with thinning set to every 10th step, a burn-in period of 2000 steps and 1000 steps for adaption. All parameters were checked for chain convergence. Autocorrelation in the MCMC chains was assessed as the effective sample size (ESS) aiming at a lower limit of 10 k for the relevant parameters. Highest density intervals (HDIs) were computed for the regression coefficients to check if coefficients were credibly non-zero. The predictors’ relative influences were assessed using standardised regression coefficients. A graph was produced by fixing all but the predictors of credible influence to their mean, resulting in a two dimensional scatter plot.

## Results

### Habitat fragmentation and habitat conditions

Our results indicated a strong decline of calcareous grasslands within the study region. The mean size of the selected grasslands patches decreased from 115,045 m^2^ in 1830–14,881 m^2^ in 2013 (Table 3). In contrast, the mean distance to the nearest grassland increased from 110 m in 1830–210 m in 2013. Confirming this observation, mean connectivity among grasslands decreased from 74.81 in 1830–27.90 in 2013. Mean loss of calcareous grasslands within the 3 km radius around each of our study sites was 78.62%.

**Table 3:** Habitat fragmentation data

St.	Name	HA_2013_	HA_1830_	HA/P	D_2013_	D_1830_	CO_1830_	CO_2013_	HL
01	Eichelberg	445	715,410	2.41	58	129	100.90	8.35	83.01
02	Münchsried	631	0	3.95	980	133	23.89	2.34	79.12
03	Oel	763	6725	3.44	391	81	9.76	1.96	61.88
04	Staudenberg	1020	0	5.31	97	70	37.16	11.65	67.04
05	Eitelberg	1399	0	8.56	117	97	130.80	44.65	84.25
06	Kühschlag	1440	3308	8.25	340	98	62.28	26.52	82.43
07	Kallmünz	1546	11,072	6.67	175	168	82.16	48.29	83.75
08	Kronbuckel	1695	1176	4.79	382	62	59.55	13.94	80.55
09	Ziegelhütte	2495	0	4.69	41	24	70.72	15.55	78.33
10	Weichseldorf	5659	37,519	12.26	290	273	43.21	9.24	71.19
11	Fuchsenbügl	6211	13,243	11.22	60	15	101.79	66.19	83.21
12	Undorf	8009	0	20.89	91	59	132.94	50.75	84.49
13	Schafbuckel	12,033	17,338	17.12	150	192	13.19	3.60	77.90
14	Goldberg	22,160	0	12.31	32	121	21.15	7.19	66.83
15	Schönhofen	21,894	58,015	23.84	211	251	78.99	32.96	81.99
16	Traidendorf	24,405	134,710	15.02	58	44	97.91	71.44	84.95
17	Gänsleite	64,984	440,768	23.17	222	63	97.09	48.36	78.88
18	Pfaffenberg	91,067	631,523	24.87	87	94	183.16	38.90	85.32
	Mean	14,881	115,045	11.60	210	110	74.81	27.90	78.62
	SE	±5828	±53,938	±1.79	±53	±17	±11.01	±5.38	±1.67

Area of the selected study sites in m^2^ in 1830 and 2013 (HA_1830_ and HA_2013_), the area/perimeter ratio (HA/P) in 2013, the distance to the nearest calcareous grassland in meter (D_1830_ and D_2013_) and the connectivity of the grasslands (CO_1839_ and CO_2013_) within a radius of 3 km in 1830 and 2013, and the loss of calcareous grasslands within this radius since 1830 in % (HL)

Habitat conditions strongly differed between study sites. Vegetation height ranged from 0.51 to 1.18 m with an average of 0.93 m (Table 4). Large differences could also be observed for the cover of grass, which varied between 48.0 and 90.0% with a mean of 76.5%. The cover of litter ranged from 7.7 to 38.0% with a mean of 20.4%, whereas the proportion of bare soil was minimum 0% and maximum 5.5% with an average value of 0.7%.

**Table 4:** Habitat condition data

St.	Name	VH	CG	CL	BS	P	K	C/N
01	Eichelberg	1.18	92.8	23.0	0.0	14.70	369.53	18.3
02	Münchsried	0.95	62.5	19.0	0.3	15.66	101.22	13.7
03	Oel	0.94	90.0	24.0	0.0	36.48	232.81	15.6
04	Staudenberg	1.54	67.0	29.0	0.0	53.76	272.77	16.0
05	Eitelberg	1.08	88.2	16.6	0.0	14.18	130.26	22.6
06	Kühschlag	0.91	87.0	30.5	0.3	26.63	192.97	42.0
07	Kallmünz	0.77	82.5	15.0	5.5	12.70	195.62	17.6
08	Kronbuckel	1.13	88.8	10.3	0.4	23.85	220.63	16.6
09	Ziegelhütte	0.93	84.5	17.5	0.8	37.63	169.02	21.8
10	Weichseldorf	0.51	63.0	14.5	0.8	16.25	135.42	20.1
11	Fuchsenbügl	1.15	74.5	19.0	0.1	31.92	249.64	19.0
12	Undorf	1.13	90.3	25.5	0.1	41.19	173.90	37.4
13	Schafbuckel	1.01	78.0	07.7	0.0	37.90	240.98	18.1
14	Goldberg	1.13	62.0	29.0	0.2	37.57	127.73	19.9
15	Schönhofen	0.43	73.0	20.5	0.2	37.63	247.30	17.6
16	Traidendorf	0.31	48.0	38.0	1.5	09.62	319.02	13.9
17	Gänsleite	0.98	66.0	17.0	1.8	20.67	294.17	10.9
18	Pfaffenberg	0.65	78.0	10.4	0.5	08.04	126.00	11.1
	Mean	0.93	76.5	20.4	0.7	26.47	211.06	19.6
	SE	±0.1	±3.0	±1.9	±0.3	±3.11	±17.52	±1.9

Habitat conditions of the selected study sites, described by the height of the vegetation in meter (VH), the cover of litter in % (CL), the cover of grass in % (CG), the proportion of bare soil in % (BS) as well as the content of phosphorous in mg/kg soil (P), potassium in mg/kg soil (K) and the ratio of carbon and nitrogen (C/N)

The content of phosphorous also varied between sites and ranged from 8.04 to 53.76 mg/kg soil with a mean of 26.47 mg/kg soil (Table 4). Similarly, the content of potassium varied between 101.22 and 319.02 mg/kg soil. On average we observed a potassium content of 211.06 mg/kg soil. Finally, we determined the carbon to nitrogen ratio, which ranged from 10.9 to 42.0 with a mean of 19.6 (Table 4).

### Genetic variation and hierarchical Bayesian multiple regression

AFLP analysis resulted in 120 fragments for *Primula veris*, 148 fragments for *Dianthus carthusianorum*, 285 fragments for *Medicago falcata*, 166 fragments for *Polygala comosa* and 192 fragments for *Salvia pratensis*. The proportion of polymorphic bands per species was 88.3% (*P. veris*), 97.4% (*D. carthusianorum*), 97.8% (*M. falcata*), 98.7% (*P. comosa*) and 94.3% (*S. pratensis*). Genetic diversity varied between species and populations. Mean genetic diversity of populations (Table 5) was highest in *M. falcata* (0.29), followed by *S. pratensis* (0.26), *P. comosa* (0.25) and *D. carthusianorum* (0.23). The lowest level of genetic diversity was observed in *P. veris* (0.18).

**Table 5:** Genetic diversity of the study species

St.	Name	*P.v.*	n	*D.c.*	n	*M.f.*	n	*P.c.*	n	*S.p.*	n
01	Eichelberg	0.23	13	0.26	15	0.39	15	0.34	15	0.35	13
02	Münchsried	0.30	14	0.35	15	0.38	15	0.33	15	0.36	15
03	Oel	0.20	15	0.26	15	0.38	15	–	–	0.35	15
04	Staudenberg	0.20	15	0.25	15	0.36	15	–	–	0.37	14
05	Eitelberg	0.25	15	0.32	15	0.36	15	0.27	15	0.34	15
06	Kühschlag	0.27	15	0.32	15	0.36	15	0.31	15	0.33	15
07	Kallmünz	0.30	15	0.28	15	0.35	15	0.29	15	0.33	15
08	Kronbuckel	0.29	12	0.30	15	0.37	15	–	–	0.37	15
09	Ziegelhütte	0.30	15	0.31	15	0.38	15	0.36	11	0.36	15
10	Weichseldorf	0.22	15	0.29	15	0.37	15	0.33	15	0.34	15
11	Fuchsenbügl	0.22	15	0.29	15	0.37	15	0.31	15	0.35	15
12	Undorf	0.22	15	0.32	15	0.37	15	0.28	15	0.33	15
13	Schafbuckel	0.30	15	0.34	15	0.38	15	0.31	14	0.35	15
14	Goldberg	0.23	15	0.35	15	0.39	15	0.35	15	0.36	15
15	Schönhofen	0.26	15	0.31	15	0.36	15	0.29	15	0.33	15
16	Traidendorf	0.24	15	0.31	15	0.36	15	0.31	15	0.32	15
17	Gänsleite	0.27	15	0.31	15	0.38	15	0.31	15	0.34	15
18	Pfaffenberg	0.32	12	0.29	13	0.37	15	0.37	15	0.36	15
	Mean/total	0.18	261	0.23	268	0.29	270	0.25	220	0.26	267
	SE	±0.01		±0.01		±0		±0.01		±0.00	

Nei’s Gene Diversity of *Primula veris* (*P.v.*), *Dianthus carthusianorum* (*D.c.*), *Medicago falcata* (*M.f.*), *Polygala comosa* (*P.c.*) and *Salvia pratensis* (*S.p.*) and the respective sample sizes

**Fig. 2:**
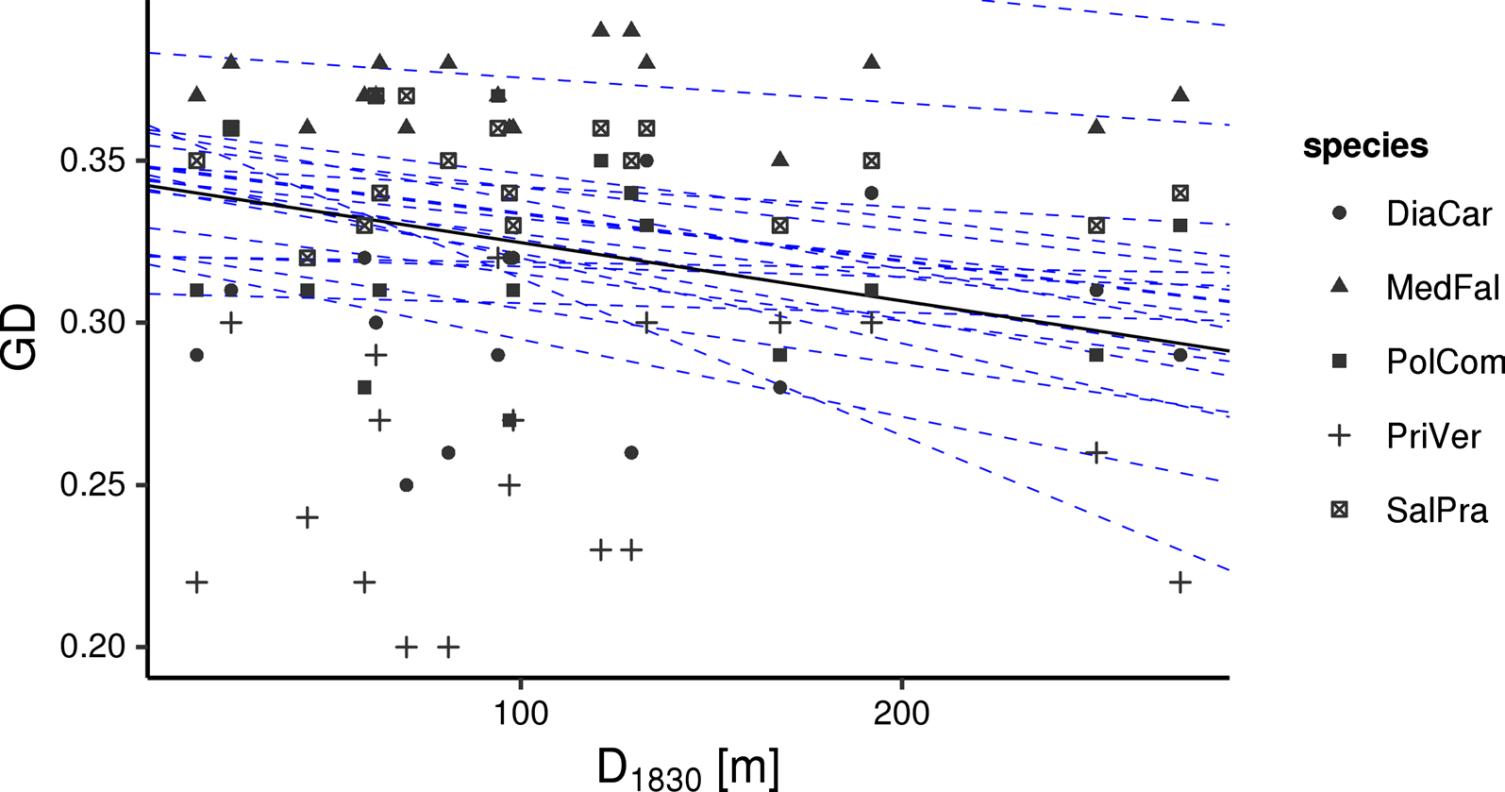
Relationship between genetic diversity (GD) and distance to the nearest calcareous grassland in 1830 (D_1830_) on 18 selected calcareous grasslands in south eastern Germany displayed as two dimensional scatter plot based upon the results of the hierarchical Bayesian multiple regression. *Dashed lines* represent twenty randomly chosen steps from the MCMC chains and are added to depict the variability in the posterior distribution of the regression parameters. Note that while intercepts vary considerably due to different levels of gene diversity in the analysed species, slopes are uniformly negative

Considering each species separately and all species together in the hierarchical Bayesian multiple regressions, we observed a credible impact of the distance to the nearest calcareous grassland in 1830 on the genetic variation within populations of the studied grassland species (Fig. 2, Tables 6, 7). The distance to the nearest calcareous grassland in 1830 was negatively correlated to the genetic variation within populations. This means that high levels of genetic variation have been detected at study sites which were closely located to other fragments in 1830. However, habitat area, today’s distance to the nearest calcareous grassland, habitat shape, habitat conditions and population size had no impact on the genetic diversity of the species.

**Table 6:** Bayesian multiple regressions for each of the analysed plant species

GV_within_	RC	ESS	Lower HDI limit	Upper HDI limit
*Primula veris*				
Intercept	−1.24	170.597	−1.44	−1.04
HA_1830_	0.16	12.534	−0.28	0.58
HA_2013_	−0.14	17.675	−0.62	0.31
HA/P	0.14	17.476	−0.29	0.58
D_1830_	*−0.28*	*22.780*	*−0.53*	*−0.04*
D_2013_	0.02	114.451	−0.14	0.25
CO_1830_	−0.08	11.826	−0.50	0.32
CO_2013_	−0.20	11.931	−0.63	0.22
P	−0.07	27.770	−0.40	0.19
K	−0.17	12.490	−0.43	0.12
C/N	0.09	35.921	−0.14	0.34
VH	−0.13	49.200	−0.43	0.08
CG	−0.11	35.207	−0.36	0.20
CL	−0.25	50.816	−0.59	−0.02
BS	0.16	57.745	−0.05	0.43
NI	0.26	104.064	−0.13	0.99
*Dianthus carthusianorum*				
Intercept	−0.31	233.219	−0.50	−0.12
HA_1830_	0.05	13.246	−0.38	0.52
HA_2013_	−0.20	18.615	−0.69	0.26
HA/P	0.31	19.995	−0.11	0.81
D_1830_	*−0.27*	*23.119*	*−0.52*	*−0.03*
D_2013_	0.00	119.211	−0.17	0.20
CO_1830_	−0.14	12.332	−0.54	0.28
CO_2013_	−0.18	12.178	−0.62	0.27
P	−0.07	29.190	−0.38	0.21
K	−0.18	13.066	−0.48	0.09
C/N	0.12	38.994	−0.10	0.40
VH	−0.09	53.816	−0.31	0.12
CG	−0.19	36.515	−0.48	0.08
CL	−0.14	50.200	−0.37	0.10
BS	0.01	43.275	−0.19	0.20
NI	0.02	87.303	−0.73	0.51
*Medicago falcata*				
Intercept	1.06	209.393	0.85	1.28
HA_1830_	0.26	12.321	−0.18	0.69
HA_2013_	−0.18	17.310	−0.66	0.27
HA/P	0.18	16.981	−0.23	0.60
D_1830_	*−0.28*	*21.349*	*−0.51*	*−0.04*
D_2013_	−0.03	114.837	−0.21	0.13
CO_1830_	−0.10	11.349	−0.51	0.29
CO_2013_	−0.19	11.427	−0.63	0.21
P	−0.02	25.143	−0.29	0.26
K	−0.13	13.169	−0.39	0.17
C/N	0.06	36.149	−0.16	0.29
VH	−0.07	55.816	−0.27	0.13
CG	−0.12	33.158	−0.36	0.12
CL	−0.13	49.603	−0.34	0.09
BS	0.01	42.573	−0.17	0.20
NI	0.01	131.943	−0.91	0.55
*Polygala comosa*				
Intercept	−0.02	99.030	−0.49	0.35
HA_1830_	0.30	13.267	−0.14	0.80
HA_2013_	−0.08	18.712	−0.53	0.42
HA/P	0.05	18.994	−0.45	0.48
D_1830_	*−0.29*	*22.677*	*−0.55*	*−0.06*
D_2013_	−0.06	120.552	−0.25	0.11
CO_1830_	−0.10	11.951	−0.53	0.30
CO_2013_	−0.28	12.171	−0.72	0.15
P	−0.02	29.128	−0.31	0.31
K	−0.14	12.706	−0.45	0.13
C/N	0.04	42.379	−0.19	0.28
VH	−0.12	45.828	−0.38	0.09
CG	−0.16	34.579	−0.45	0.10
CL	−0.14	56.817	−0.36	0.10
BS	0.02	46.885	−0.18	0.20
NI	0.04	90.504	−1.45	1.05
*Salvia pratensis*				
Intercept	0.55	171.962	0.34	0.76
HA_1830_	0.20	12.550	−0.24	0.62
HA_2013_	−0.20	20.218	−0.75	0.33
HA/P	0.12	17.543	−0.34	0.51
D_1830_	*−0.29*	*22.443*	*−0.52*	*−0.05*
D_2013_	−0.03	111.415	−0.20	0.13
CO_1830_	−0.10	11.783	−0.50	0.31
CO_2013_	−0.19	11.411	−0.63	0.21
P	0.02	27.334	−0.27	0.31
K	−0.13	12.997	−0.40	0.15
C/N	0.03	40.560	−0.20	0.26
VH	−0.04	63.303	−0.24	0.20
CG	−0.14	32.465	−0.37	0.11
CL	−0.16	49.189	−0.38	0.05
BS	0.01	44.441	−0.17	0.20
NI	0.02	90.645	−0.18	0.24

Results of the Bayesian multiple regressions on genetic variation within populations (GV_within_) calculated on species-dependent level. Modal values of marginal distributions of each standardised regression coefficient are given together with the effective sample size (ESS) of all parameters. A 90% highest density interval (HDI) was computed for each model parameter. The distance to the next calcareous grassland in 1830 (D_1830_) exhibits a credible impact on the genetic variation of the selected species (in italic letters) as its HDI excludes zero (*RC* standardised regression coefficient)

**Table 7:** Hierarchical Bayesian multiple regression

GV_within_	RC	ESS	Lower HDI limit	Upper HDI limit
Intercept	0.01	300.000	−1.47	1.46
HA_1830_	0.20	13.665	−0.34	0.75
HA_2013_	−0.16	18.771	−0.74	0.40
HA/P	0.15	20.222	−0.39	0.70
D_1830_	*−0.29*	*22.242*	*−0.57*	*<0.00*
D_2013_	−0.02	130.088	−0.23	0.20
CO_1830_	−0.09	11.912	−0.60	0.37
CO_2013_	−0.21	11.813	−0.73	0.30
P	−0.03	29.774	−0.38	0.31
K	−0.15	12.880	−0.48	0.19
C/N	0.07	40.678	−0.20	0.36
VH	−0.10	58.448	−0.37	0.17
CG	−0.15	36.036	−0.46	0.17
CL	−0.16	62.511	−0.46	0.12
BS	0.04	59.163	−0.21	0.31
NI	0.02	164.573	−0.96	0.87
Variance parameter	0.44	35.412	0.24	0.58
Normality parameter	4.45	7044	1.00	82.89

Results of the hierarchical Bayesian multiple regression on genetic variation within populations (GV_within_) calculated on species-independent level. Modal values of marginal distributions of each standardised regression coefficient are given together with the effective sample size (ESS) of all parameters. A 95% highest density interval (HDI) was computed for each model parameter. The distance to the next calcareous grassland in 1830 (D_1830_) exhibits a credible impact on the genetic variation of all species at the selected study sites (in italic letters) as HDI <0 (*RC* standardised regression coefficient)

## Discussion

### Impact of habitat fragmentation on genetic diversity

In our study, we observed an impact of the historical landscape configuration on the genetic diversity of the study species, since it depended on the distance to the nearest calcareous grassland in 1830 in the hierarchical Bayesian regressions. During the process of fragmentation the area of the habitat patches usually decreases [8]. Consequently, the size of plant populations occurring in remnant calcareous grasslands also declines, which results in a decline in genetic diversity [10]. In our study, we also observed a strong reduction of the habitat size. The contemporary area of the study sites was on average only 10% of the area in 1830. However, we observed no significant relationship between genetic diversity of the five study species and population size. Neither the current habitat area, nor the actual number of individuals per grassland fragment had an impact on genetic diversity. Similar findings have been reported in other fragmentation studies [12, 42]. Lag effects, a delayed reaction of genetic diversity on the reduction of population size [43], which is comparable to the extinction debt reported for species diversity [44, 45], could be a reason for the observed lack of a relationship between genetic diversity and population size [46]. In this case, genetic diversity should then be related to the historical area of the study sites. However, our study provided no evidence for such a relationship, which means that genetic diversity may generally be determined by factors other than habitat area or population size. Stochastic gene flow and long term survival under highly fragmented conditions are often considered as reasons for this observation [9, 47]. It has also been stated that the absence of this relationship may occur when the habitat area rapidly changes relative to the generation time of the study species [15]. Indeed, the calcareous grasslands in this study were formerly widely distributed and may have exhibited more or less erratic gene flow due to grazing. In combination with the long term persistence of the grassland species [48] these factors may be the most likely explanation for the lack of relationship between habitat area or population size and genetic diversity in this study.

Aside from decreasing habitat area, isolation of calcareous grasslands is also an important consequence of habitat fragmentation [7, 8]. The continuous process of increasing isolation affects genetic variation both between and within populations since gene flow by seeds and pollen declines with increasing isolation [8]. As gene flow decreases, the effects of genetic drift and inbreeding are intensified [49]. This results in an increased level of genetic variation between populations and a progressive loss of genetic variation within populations [50].

Gene flow by pollen is normally restricted to the nearest vicinity of plant populations to distances of less than 1 km [51, 52]. However, rare pollination events may also allow gene flow over larger distances [53]. Therefore, gene flow among fragmented calcareous grasslands at a larger scale is mainly caused by endo- and ectozoochorous seed dispersal, especially from migration of sheep flocks [2]. It has been shown, that the genetic structure of plant populations depends on present landscape connectivity [54] and that genetic variation between populations is affected by geographic distance between populations [8]. Moreover, it has also been demonstrated that genetic diversity may depend on habitat age both in natural [55] and semi-natural [56] habitats. In contrast to previous studies, which reported higher levels of genetic diversity in populations from historically older habitat fragments of forests [57] or calcareous grasslands [58], we observed no impact of habitat age on genetic diversity in our analysis.

Surprisingly, neither historical nor present habitat connectivity had an impact on genetic diversity in our study. This may be traced back to the fact that the calculated connectivity reflects the spatial structure, but not the real gene flow, which may be strongly affected by the migration of sheep flocks [46]. However, we observed a relationship between genetic diversity of the grassland species and the distance to the nearest grassland patch in 1830. Therefore, the historical landscape configuration is more important for the genetic diversity of calcareous grassland species than the present landscape structure. The effect of historical landscape configuration on species diversity has so far been demonstrated in several studies [59, 60], whereas the impact on genetic diversity has scarcely been shown [27]. However, it has recently been reported that the genetic diversity of the grassland species *Succisa pratensis* depends on the historical landscape structure of the habitat [15], which supports the results of our study. Moreover, it has been shown that the genetic diversity of *Dianthus carthusianorum* depends on patch connectivity by shepherding [61] and that population disconnection can create a genetic bottleneck, even in the absence of a demographic collapse [62], which underlines the importance of historical gene flow for the level of current genetic diversity.

### Impact of habitat conditions on genetic variation

The results of our analyses indicated no impact of habitat conditions on genetic diversity of the studied calcareous grassland species. It has been demonstrated that, alongside habitat fragmentation, changes in habitat conditions have a strong impact on the species richness and composition of remnant calcareous grasslands fragments [18, 21]. Lack of grazing and the accumulation of soil nutrients lead to the loss of the typical open short-grass vegetation structure, allowing for the existence of many less competitive herbs. Under the conditions of abandonment and due to increased levels of nutrients, grasses such as *Brachypodium pinnatum* become increasingly dominant [19] and litter accumulates [63]. As a consequence, species requiring light for germination decline due to the effects of ground shadowing [24]. For the calcareous grasslands studied here, it has been demonstrated, that species diversity strongly depends on vegetation height and litter cover. Lack of grazing is therefore the most important reason for the declining species diversity of the grasslands, whereas fragmentation aspects play no significant role.

In contrast to the impact of land use on species diversity, the relationship between land use and genetic diversity is much less clear. However, it has already been demonstrated, that seedling recruitment and establishment in grasslands are positively affected by grazing and the removal of litter [64]. Schleuning et al. [65] even stated that the grassland species *Trifolium montanum* is more threatened by the effects of habitat degradation in the short term, than by the effects of fragmentation. Since genetic diversity depends explicitly on the degree of sexual reproduction [66], it appears possible that habitat conditions may also have an effect on genetic diversity. For the grassland species *Dianthus seguieri*, it has recently been shown that increased vegetation height and coverage as well as a high proportion of graminoids due to land use abandonment reduce genetic diversity and seed set [67]. As previously reported, land use by grazing generally has a positive impact on genetic diversity [68] since it reduces the cover of litter, and therefore may stimulate sexual reproduction.

However, in this study genetic diversity depended neither on vegetation structure, nor on soil nutrient levels. One reason for this observation may lie in the life span of the investigated plant species. It has already been reported that the frequency of plant species in remnant calcareous grasslands depends mainly on their persistence [48]. All species included in this study are long-lived perennials [69] and since the process of habitat deterioration due to abandonment goes back only about 50 years, many individuals we analysed may have been established before litter accumulation reached a critical level. This means that impaired habitat conditions may not yet have resulted in decreased levels of genetic diversity. Another reason for the lack of relationship between habitat conditions and genetic diversity could be the persistence of seeds in the soil seed bank, which may have contributed to the regeneration of the populations and to the maintenance of genetic diversity within the studied populations [70].

## Conclusions

The results of our study provide evidence that the genetic diversity of calcareous grassland plant species depends on historical landscape configuration, rather than on the present population size or habitat conditions. In practice, efforts to preserve calcareous grasslands mainly concentrate on large fragments exhibiting the typical open-shortgrass habitat conditions and high species diversity. However, a comprehensive conservation approach should also consider the genetic diversity of calcareous grassland plant species. From our results it can be concluded that populations in smaller grassland fragments may, depending on historical landscape configuration, substantially contribute to the genetic variation of the plant species even under conditions of habitat deterioration. Preferably, these populations should therefore be included in strategies to preserve calcareous grasslands as the local biodiversity hotspots they are.

## Additional file

**Additional file 1.** Text file containing the specification for the JAGS model used for the Bayesian hierarchical multiple regression. The basic model definition was adapted from Kruschke [40].
